# Supplementary material for: N-acetyl-aspartate and Myo-inositol as Markers of White Matter Microstructural Organization in Mild Cognitive Impairment: Evidence from a DTI-1H-MRS Pilot Study
Source: Diagnostics (Basel). 2023 Feb 9;13(4):654. doi: 10.3390/diagnostics13040654 (PMC9955118; doi:10.3390/diagnostics13040654)
Supplement: Supplementary file 1 [file diagnostics-13-00654-s001.zip › diagnostics-2183488-supplementary.pdf]

Supplementary S1: MRSinMRS Reporting Checklist for single-voxel MR spectroscopy imaging[1]

1. Hardware
   a. Field strength: 3T
   b. Manufacture: Siemens
   c. Model: Skyra (VE11E)
   d. RF coil: 32 channels ^{1}H- head coil

2. Acquisition
   a. Pulse sequence: PRESS
   b. Volume of interest (VOI) locations:
      i. dorsal posterior cingulate cortex (dPCC)
      ii. left primary sensorimotor cortex (left SM1)
      iii. left hippocampus (left HPC)
      iv. left medial temporal cortex (left MTC)
      v. right dorsolateral prefrontal cortex (right dlPFC)
   c. Nominal VOI size:
      i. 16 × 16 × 16 mm^3 (dPCC, left SM1, right dlPFC)
      ii. 20 × 12 × 16 mm^3 (left MTC)
      iii. 26 × 12 × 12 mm^3 (left HPC)
   d. Repetition time (T_R) and echo time (T_E)
      i. T_R = 2000 ms,
      ii. T_E = 30 ms
   e. Total number of acquisitions per spectrum: 128 averages
   f. Spectral bandwidth: 2000 Hz
   g. Number of spectral points: 1024 points
   h. Water suppression method: CHESS (bandwidth = 50 Hz)
   i. Shimming method: Automated B0-field mapping followed by manual adjustment to reduce water signal FHWM below 15Hz

3. Analysis software and outputs
   a. Analysis software: LCModel 6.3.1-R
   b. Output measures: Ratio to total creatine (tCr) of tNAA, tCho, mIns, and Glx
   c. Processing steps: estimation of water-referenced values of total NAA (tNAA = NAA + NAAG), total choline (tCho = GPC + PCh), mIns, and glutamate-glutamine complex (Glx = Glu + Gln)

4. Fitting model basis set
   27 basis spectra including: alanine (Ala), aspartate (Asp), creatine (Cr), phosphocreatine (PCr), γ-aminobutyric acid (GABA), glucose (Glc), glutamine (Gln), glutamate (Glu), glycerophosphocholine (GPC), phosphorylcholine (PCh), myo-inositol (mIns), lactate (Lac), N-acetyl aspartate (NAA), N-acetyl-aspartyl-glutamate (NAAG), scyllo-Inositol (Scyllo), taurine (Tau), negative creatine methylene (-CrCH2), guanidinoacetate (Gua), lipids [Lip09, Lip13a, Lip13b, and Lip20] and macromolecules [MM09, MM12, MM14, MM17 and MM20]

5. Data quality
   a. Data exclusion criteria: FHWM > 15 Hz, signal to noise ratio (SNR) < 5, or Cramér-Rao lower bound (CRLB) > 20 %
   b. Reported measures of SNR and FWHM (in ppm) as reported by LCModel for included spectra:

i. dPCC (57 spectra), SNR [16.2 ± 3.10 (10-24)], FWHM [0.062 ± 0.016 (0.033 – 0.105) ppm]
ii. left SM1(59 spectra), SNR [21.2 ± 4.60 (11-32)], FWHM [0.069 ± 0.016 (0.033 – 0.095) ppm]
iii. left HPC (51 spectra), SNR [8.61 ± 1.70 (6-12)], FWHM [0.075 ± 0.015 (0.043 – 0.105) ppm]
iv. left MTC (47 spectra), SNR [11.0 ± 2.42 (5-17)], FWHM [0.071 ± 0.014 (0.043 – 0.095) ppm]
v. right dlPFC (57 spectra), SNR [18.1 ± 4.03 (10-26)], FWHM [0.076 ± 0.020 (0.033 – 0.105) ppm]

c. CRLB as reported by LCModel for included spectra:
   i. dPCC: tNAA [3.74 ± 0.74 (2-6) %], tCho [4.72 ± 0.70 (3-6) %], mIns [5.46 ± 1.25 (4-9) %], Glx [7.07 ± 1.64 (5-13) %], tCr [3.04 ± 0.50 (2-4) %]
   ii. left SM1: tNAA [3.05 ± 0.88 (2-5) %], tCho [3.51 ± 0.57 (2-5) %], mIns [5.34 ± 1.09 (3-9) %], Glx [11.1 ± 2.34 (6-17) %], tCr [2.83 ± 0.50 (2-4) %]
   iii. left HPC: tNAA [5.83 ± 1.19 (3-8) %], tCho [4.80 ± 0.96 (3-7) %], mIns [5.25 ± 1.23 (3-9) %], Glx [9.71 ± 2.55 (6-19) %], tCr [4.37 ± 0.77 (3-6) %]
   iv. left MTC: tNAA [5.06 ± 1.39 (3-8) %], tCho [4 ± 1.0 (3-7) %], mIns [5.66 ± 1.74 (3-11) %], Glx [8.49 ± 2.18 (6-16) %], tCr [3.89 ± 0.87 (3-6) %]
   v. right dlPFC: tNAA [3.30 ± 1.00 (2-6) %], tCho [3.40 ± 0.56 (3-5) %], mIns [4.56 ± 0.96 (3-10) %], Glx [10.3 ± 2.72 (7-18) %], tCr [2.90 ± 0.59 (2-4) %]

[1]In line with the guidelines presented in Lin A, Andronesi O, Bogner W, et al. Minimum Reporting Standards for in vivo Magnetic Resonance Spectroscopy (MRSinMRS): Experts' consensus recommendations. NMR in Biomedicine 2021; 34: e4484.
